# Changes in choroidal vessel morphology associated with fluid leakage in central serous chorioretinopathy; a comparison of OCTA and ICGA

**DOI:** 10.1007/s00417-025-06918-2

**Published:** 2025-08-06

**Authors:** Payam Kabiri, Steffen Künzel, Shirin Ashraf-Vaghefi, Theresa Bonaventura, Anne Rübsam, Antonia Joussen, Oliver Zeitz

**Affiliations:** Department of Ophthalmology, Charité Universitätsmedizin Berlin, Campus Benjamin Franklin, Corporate Member of Freie Universität Berlin, Humboldt-Universität zu Berlin and Berlin Institute of Health, Hindenburgdamm 30, 12203 Berlin, Germany

**Keywords:** Central serous chorioretinopathy, Choroidal imaging, Intervortex venous anastomoses, Pachychoroid, Pachyvessels

## Abstract

**Purpose:**

To identify changes in choroidal morphology crucial for CSCR this study assessed the relationship between pachychoroidal changes and the occurrence of CSCR. Additionally, given the undefined role of optical coherence tomography angiography (OCTA) in CSCR diagnosis, this study evaluated OCTA as an alternative to indocyanine green angiography (ICGA).

**Methods:**

Multimodal imaging of 99 patients affected by unilateral or bilateral CSCR was retrospectively assessed to identify pachychoroidal changes. Through logistic regression the relationship between these choroidal changes and CSCR occurrence was examined. Evaluation of ICGA and OCTA images was conducted separately, followed by a comparative analysis of the obtained results.

**Results:**

Presence of intervortex venous anastomoses significantly increased the likelihood of CSCR affection (*P* = 0.0004, odds ratio 4.829), whereas presence of pachyvessels did not (*P* = 0.7947). Comparative evaluation of pachychoroidal changes between OCTA and ICGA images resulted in 95.16% of cases in coinciding results. OCTA images exhibited less impairment in quality due to subretinal fluid than ICGA images.

**Conclusion:**

Intervortex venous anastomoses were significantly associated with the presence of subretinal fluid and serous pigment epithelium detachment; however, longitudinal studies are required to establish a causal relationship. OCTA has emerged as a non-invasive alternative to ICGA to assess pachychoroidal changes in patients with CSCR.

## Key messages

*****What is known***:**

- Central serous chorioretinopathy (CSCR) is a pachychoroidal disease entity.
- Pachychoroidal changes include increased subfoveal choroidal thickness, dilation of choroidal veins in Haller’s layer (pachyvessels), and prominent choroidal vessels crossing watershed zones that divide vortex vein territories.

*****What is new***:**

- While pachyvessels and increased subfoveal choroidal thickness are common in both eyes with CSCR activity and fellow eyes without CSCR activity, the collapse of the central watershed due to intervortex venous anastomoses is significantly associated with the presence of subretinal fluid or serous retinal pigment epithelium (RPE) detachment.
- OCTA scans with Haller’s layer segmentation provide additional insights into the vascular involvement in manifest CSCR with less impairment through subretinal fluid.

## Introduction

Central serous chorioretinopathy (CSCR) was the first described disease entity within the pachychoroid disease spectrum [1]. The pachychoroidal disease spectrum encompasses a heterogeneous group of diseases that share the eponymous characteristic of a thickened choroid, known as pachychoroid [2–4]. A subfoveal total choroidal thickness (SFT) exceeding 300 μm defines the most common threshold for pachychoroid diagnosis [4–6]. Establishing a definitive threshold of a thickened choroid is complicated through the influence of various factors (such as blood pressure, axial bulbus length, age, etc.) on choroidal thickness [5]. Other conditions such as uveitis may also cause a thickened choroid; however, due to their distinct etiology, they are not considered part of the pachychoroidal spectrum [5].

In cases of pachychoroid, alongside the increased overall thickness of the choroid, there is dilation of the choroidal veins of the Haller’s layer, with concurrent atrophy of the Sattler’s layer and the overlying choriocapillaris [7]. The enlarged choroidal vessels of the Haller’s layer, lacking thinning towards the posterior pole of the eye, are known as pachyvessels [2, 8]. These pachyvessels are commonly situated within the thickened choroid region, often coinciding with the region of disease manifestation, resulting in the complete picture of the pachychoroid, characterized by increased overall choroidal thickness, atrophic choriocapillaris and Sattler’s layer, along with a thickened Haller layer containing pachyvessels [7, 8].

The territorial choroidal blood drainage into the vortex veins is delineated by horizontal and vertical watershed boundaries, dividing the choroid into 4 to 6 quadrants. The central venous watershed zone, passing horizontally through the optic disc and macula, delineates the boundary between the temporal-superior and the temporal-inferior vortex vein territories [9–11]. Prominent choroidal vessels, establishing connections between these different territories by crossing the watershed zones, have been described in various disease entities of the pachychoroid spectrum and are referred to as intervortex venous anastomoses [12–14].

The current hypothesis regarding the not fully understood pathogenesis of CSCR is based on the theory of venous overload: An obstructed transscleral outflow results in an elevated hydrostatic pressure within the choroidal vessels, dilation of the vessels within the Haller’s layer, and subsequently leading to fluid leakage [6, 9, 15]. The influence of systemic factors on CSCR is proven [10, 16, 17]. However, CSCR occurs more often unilaterally than bilaterally, although pachychoroidal changes (such as pachyvessels and increased SFT) have also been observed in unaffected fellow eyes without CSCR [1].

The first objective of this study was to determine the crucial changes in choroidal vessel morphology associated with subretinal fluid or serous RPE detachment by comparing the occurrence of pachychoroidal changes in eyes affected by CSCR to their fellow eyes without any signs of CSCR.

The retinal pigment epithelium (RPE) impairment in eyes with CSCR is presumed to arise from alterations of the underlying choroid [6]. A potential RPE defect can be visualized by the typical leakage point using Fluorescein Angiography (FA) [2, 18, 19]. Indocyanine Green Angiography (ICGA) is considered the standard for visualizing choroidal vessels today [6]. Hyperpermeability of the choroidal vessels during the mid-phase of ICGA corresponding to the site of disease activity has been shown even in the absence of a corresponding leakage point on FA [2, 8, 19]. This supports the assumption that the primary cause of CSCR is dysfunction of the choroid, leading to RPE damage, rather than the other way around [6]. In contrast to dye-based angiography, Optical Coherence Tomography Angiography (OCTA) does not play a decisive role in the diagnosis of CSCR [6, 20].

The second objective of this study was to assess OCTA as a non-invasive alternative to ICGA for the visualization of choroidal vessel morphology in the diagnosis of central serous chorioretinopathy (CSCR).

## Methods

In this retrospective study 198 eyes of 99 patients were included from the BIOCHOR cohort of the Berlin Macula Registry, a clinical study database. The prospectively recruited BIOCHOR cohort consists of 113 patients with acute and chronic CSCR who presented at Charité Universitätsmedizin Berlin, Germany between June 2021 and February 2023. 24 patients from the BIOCHOR cohort were excluded from this study due to missing choroidal vascular imaging (no ICGA or OCTA imaging available). Patient parameters such as age (years), sex (male; female), previous treatment, best-corrected visual acuity (BCVA) and refractive error (spherical equivalent) for all included patients were recorded at the time point of inclusion. After objective refraction (Nidek Co, USA) subjective refraction was performed by an ophthalmologist to determine the BCVA (logMAR) and the refractive error (dpt). All 198 included eyes were examined using enhanced depth imaging Optical Coherence Tomography (EDI-OCT) (Spectralis HRA + OCT, Heidelberg Engineering).

Eyes with current subretinal fluid with or without serous pigment epithelium detachment or history of subretinal fluid were defined as eyes affected by CSCR. Eyes with no signs of subretinal fluid, serous PEDs and no history of subretinal fluid or serous PEDs were defined as unaffected fellow eyes.

Additionally, based on the EDI-OCT images, the subfoveal choroidal thickness (SFT) was measured as the perpendicular distance between Bruch’s membrane and the choroidoscleral border, encompassing all choroidal layers (Choriocapillaris, Sattler, and Haller layer) using the caliper tool provided in the Heidelberg Eye-Explorer. A SFT of 300 μm was considered the threshold for defining a thickened choroid as commonly reported in the literature, hereafter referred to as pachychoroid [4–6]. Axial length data was not available for the cohort.

The Indocyanine Green Angiography (ICGA) was conducted to visualize the choroidal vessels and their bloodflow in 134 out of the 198 included eyes. Immediately after the injection of 2.5 ml of Verdye (5 mg/ml) the ICGA images were captured (HRA + OCT by Heidelberg Engineering) repeatedly at intervals of every 10 to 20 s during the early phase (up to 3 min after injection), every 1 to 5 min during the middle phase (3 to 15 min after injection), and every 3 to 5 min during the late phase (over 15 min after injection) up to 25 min after dye injection. The ICGA assessment in this study focused on the ICGA images captured during the early phase (up to 3 min after injection) and the middle phase (3 to 15 min after injection).

A 6 × 6 mm Optical Coherence Tomography Angiography (OCTA) of the macula was performed on 188 out of the 198 included eyes using the Cirrus 6000 (Carl Zeiss Meditec Inc). Using the en-face analysis of the Cirrus Viewer, the choroidal vessels of the Haller’s layer were highlighted in the choroidal slabs.

**Fig. 1:**
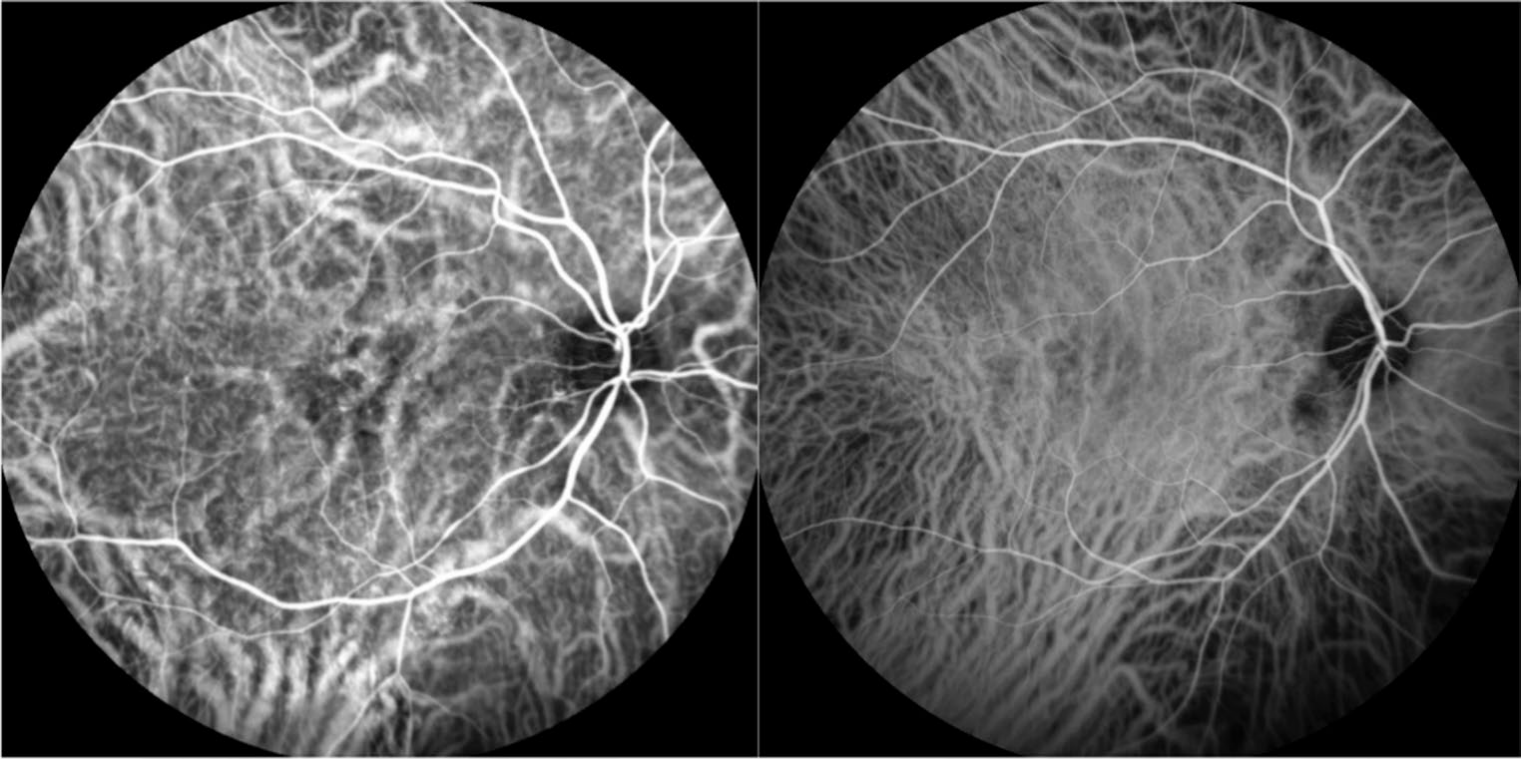
Middle phase ICGA images of an eye with active CSCR. ICGA images of the middle phase depicting choroidal pachyvessels (thickened choroidal vessels without thinning towards the posterior pole) as well as choroidal intervortex venous anastomoses (choroidal vessels crossing the central watershed between the optic disc and fovea)

**Fig. 2:**
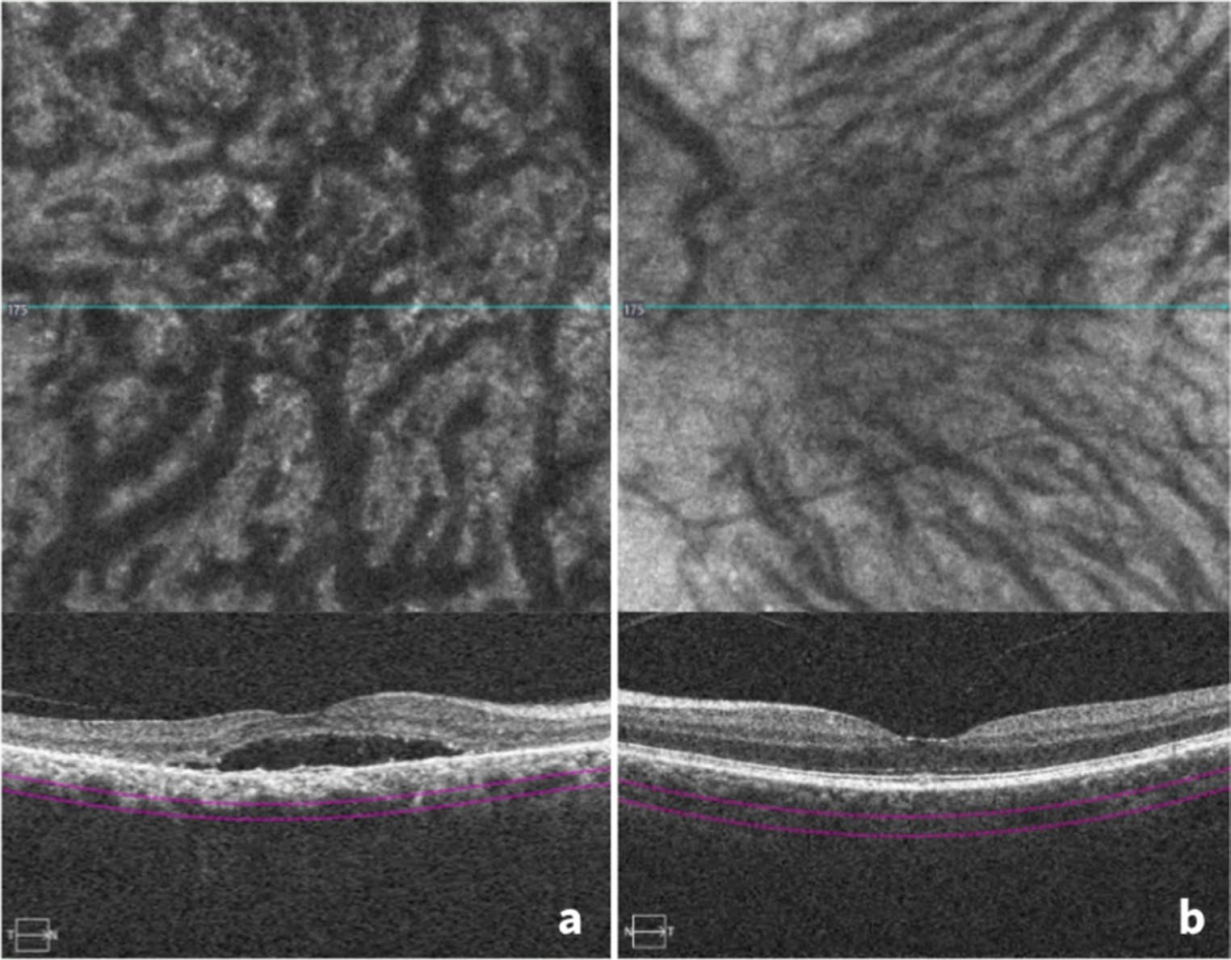
OCTA scans with highlighted Haller’s layer of an eye with active CSCR (**a**) and an eye without active CSCR (**b**). (**a**) depicts pachyvessels and intervortex venous anastomoses. (**b**) depicts neither pachyvessels nor intervortex venous anastomoses

Based on the obtained ICGA and OCTA images, the presence of pachyvessels and choroidal intervortex venous anastomoses at the central watershed was retrospectively assessed. The ICGA and OCTA images were used in conjunction to improve reliability regarding the assessment of the pachychoroidal changes, particularly in cases where either modality showed image quality limitations. Pachyvessels were defined as choroidal vessels with enlarged lumen and lack of thinning towards the posterior pole of the eye. Intervortex venous anastomoses were defined as prominent choroidal vessels crossing the central watershed from the nasal edge of the optic disc to the fovea, thereby connecting the temporal-superior and the temporal-inferior vortex vein territory (Figs. 1 and 2). Dynamic ICGA data was not available, so the identification was based on anatomical imaging alone.

The presence of pachyvessels, and intervortex venous anastomoses were recorded as binary (present/not present) for all 198 eyes.

The results are displayed as arithmetic mean +/- standard deviation of the mean if not stated otherwise. The Mann-Whitney-U test was employed to compare the age distribution between the included male and female patients, as well as to assess the visual acuity (BCVA) discrepancy between eyes affected by CSCR and the unaffected fellow eyes, both in the entire patient cohort and in separate analyses based on gender. Additionally, this test was employed to evaluate the disparity in refractive error (spherical equivalent) between eyes affected by CSCR and the unaffected fellow eyes in the entire cohort. The Wilcoxon signed-rank test was performed to compare the refractive error between the eye affected by CSCR and the unaffected fellow eye in patients diagnosed with unilateral CSCR. The Fisher’s exact test was employed to compare the occurrence rates of bilateral and unilateral CSCR among the included male and female patients. Logistic regression was performed to investigate the association between the occurrence of CSCR and the subfoveal choroidal thickness (SFT), the presence of pachyvessels, or intervortex venous anastomoses at the central watershed.

Out of the 198 eyes included a total of 124 eyes were examined using both OCTA and ICGA. The OCTA and ICGA images were separately evaluated for the presence of pachyvessels and intervortex venous anastomoses. Following this first assessment, the result of the evaluation of the OCTA image for each eye was compared with the evaluation of the ICGA image for the same eye. A match occurred when there was consensus in the assessment of the presence of pachyvessels and intervortex venous anastomoses. If the assessment of pachyvessels and intervortex venous anastomoses of an eye in OCTA and ICGA images did not correspond, it was considered a mismatch. In case of a mismatch a qualitative assessment of the image quality was performed regarding visualization of choroidal vessel continuity, image sharpness and consensus grading between two specialists. The aim was to determine which imaging modality (OCTA or ICGA) provided better image quality for the eye, thereby likely resulting in the correct assessment of the choroidal vessel morphology.

A P-value ≤ 0.05 was considered statistically significant. All statistical analyses were performed using GraphPad Prism, Version 10, for MacOS (Graphpad Software Inc., Boston, USA). Graphs were created using GraphPad Prism, Version 10, for MacOS (Graphpad Software Inc., Boston, USA). Tables were designed using Word (Microsoft Corporation, Redmond, USA).

## Results

This cohort (*n* = 99) included 77 men and 22 women (male-to-female ratio 3.5:1) with a mean age of 51.37 ± 10.87 years. The mean age of the male patients (49.05 years +/- 9.76 years) was significantly lower than the mean age of the female patients (59.5 years +/- 10.63 years) (*P* = 0.0003). Of the total cohort, 47 patients presented with acute CSCR, whereas 52 patients had previously received treatment in the context of chronic CSCR. Among these, 26 had been treated with oral acetazolamide, and 12 with oral aldosterone antagonists. No patient had a history of systemic or local corticosteroid therapy. Local ocular treatments included photodynamic therapy (PDT) in 7 eyes, focal leakage-point laser treatment in 18 eyes, topical nepafenac therapy in 26 eyes, and intravitreal injections in 22 eyes. Among the 99 patients, 56 patients (42 men and 14 women) had unilateral CSCR, while 43 patients (35 men and 8 women) were affected by bilateral CSCR. Unilateral CSCR was observed more frequently in this cohort than bilateral CSCR, for both men and women. There was no significant difference between men and women regarding the occurrence of bilateral or unilateral CSCR (*P* = 0.476). A total of 142 eyes affected by CSCR and 56 fellow eyes without signs of CSCR activity were evaluated. The mean BCVA of the unaffected fellow eyes (logMAR − 0.024 +/- 0.08) was significantly better than the eyes with CSCR (logMAR 0.28 +/- 0.39) (*P* < 0.0001). In the gender-separate analysis, the eyes of women affected by CSCR (logMAR 0.43 +/- 0.46) had a significantly lower BCVA than the eyes of men affected by CSCR (logMAR 0.23 +/- 0.36) (*P* = 0.0107). The BCVA of the unaffected fellow eyes of men (logMAR − 0.029 +/- 0.081) and women (logMAR − 0.007 +/- 0.107) was not significantly different (*P* = 0.2284).

The refractive error (spherical equivalent of the subjective refraction, dpt) of all the eyes with CSCR (0.37 +/- 2.05) was not significantly different from all the fellow eyes without CSCR (0.12 +/- 1.85) (*P* = 0.346). Among the patients with unilateral CSCR, there was also no significant difference in refractive error between the eyes with CSCR (0.23 +/- 2.02) and their fellow eyes without CSCR (0.11 +/- 1.95) (*P* = 0.1678).

The SFT of eyes affected by CSCR (400.3 μm +/- 93.88 μm) was significantly higher than the SFT of unaffected fellow eyes (354.9 μm +/- 92.35 μm) (*P* = 0.001). In 87.85% of the eyes with CSCR and in 75% of the fellow eyes without CSCR, the SFT exceeded the cutoff of 300 μm for pachychoroid (Table 1).

**Table 1:** Pachychoroidal changes in eyes with CSCR and unaffected fellow eyes

	Pachychoroid	Pachyvessels	Intervortex venous anastomoses
Eyes with CSCR (142)	87.85% (123)	93.66% (133)	92.96% (132)
Unaffected fellow eyes (56)	75% (42)	94.64% (53)	73.21% (41)

Thus, a pachychoroid was present in most of the eyes with CSCR as well as the unaffected fellow eyes without any sign of CSCR on EDI-OCT. In 12.15% of the eyes with CSCR and in 25% of the unaffected fellow eyes, pachychoroid was absent. The probability of CSCR with subretinal fluid or pigment epithelium detachment increased by 0.5% with each micrometer increase in SFT (odds ratio (OR) 1.005, *P* = 0.0033) (Table 2).

**Table 2:** Logistic regression analysis of CSCR presence and pachychoroid markers

	Significant correlation to CSCR?	Odds Ratio
Subfoveal choroidal thickness in µm	*P* = 0.0033	1.005
Presence of pachyvessels	*P* = 0.795	0.837
Presence of intervortex venous anastomoses	*P* = 0.0004	4.829

In over 90% of eyes, both with CSCR (93.66%) and the fellow eyes without signs of CSCR activity in OCT (94.64%), pachyvessels were present (Table 1). Therefore, the presence of pachyvessels did not significantly increase the likelihood of CSCR affection (*P* = 0.795, OR 0.837) (Table 2).

Intervortex venous anastomoses of the choroidal vessels at the central watershed were more frequently observed in eyes with CSCR (92.96%) compared to the fellow eyes without CSCR (73.21%) (Table 1). The presence of these intervortex venous anastomoses showed a significant correlation to the presence of CSCR (*P* = 0.0004, OR 4.829) (Table 2).

**Fig. 3:**
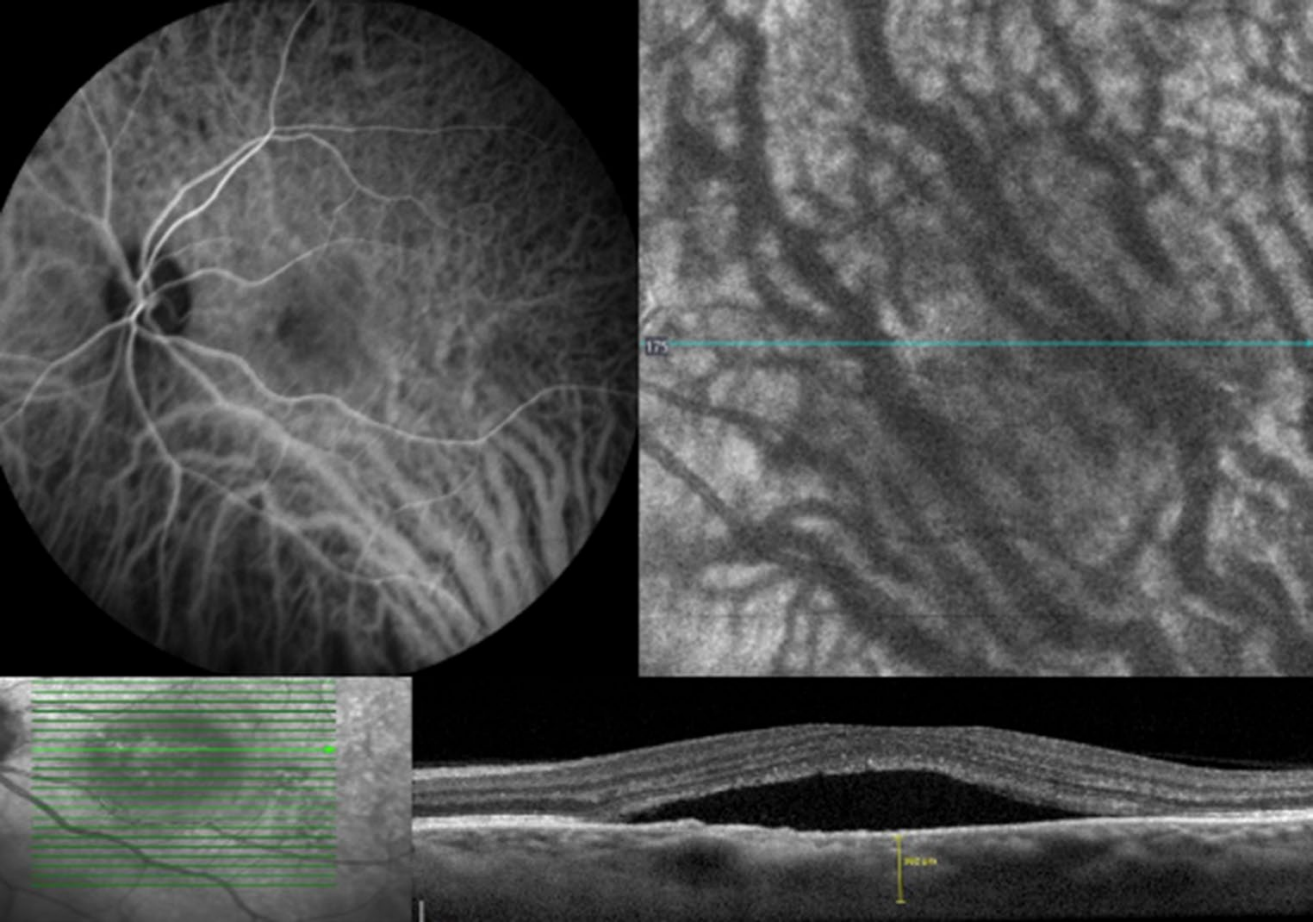
Multimodal imaging of an eye with manifest CSCR. The ICGA image exhibits a restricted image quality within the fovea. The choroidal vascular morphology at the central watershed is not clearly assessable. The corresponding OCTA scan of the fovea and temporal parapapillary region clearly depicts the choroidal intervortex anastomoses, crossing the central watershed. The OCT-EDI scan of the macula shows foveal subretinal fluid, which impairs the image quality of the ICGA, while the OCTA depicts the deep choroidal vessels with good image quality

In the separate evaluation of the OCTA and ICGA images, a match in the assessment of the images regarding the presence of pachyvessels and intervortex venous anastomoses was observed in 95.16% of the 124 eyes examined using both imaging modalities. In the remaining 4.84% of eyes with mismatched assessments of intervortex anastomoses and pachyvessels OCTA was considered superior in all such cases (6/6) regarding the presence of intervortex venous anastomoses and 4 out of 6 cases regarding the presence of pachyvessels based on better visualization of choroidal vessel continuity, reduced masking by subretinal fluid, and higher image sharpness at the central watershed zone (Fig. 3).

## Discussion

Increasing subfoveal choroidal thickness (SFT) was significantly associated with an increased likelihood of CSCR affection, raising the probability of CSCR by 0.5% per micron increase in SFT. This finding supports the potential role of choroidal thickness and the pachychoroid in the pathogenesis of this condition. Although the SFT demonstrated a significant correlation to the probability of CSCR affection, 12.15% of eyes affected by CSCR did not exhibit a choroidal thickness exceeding the cutoff of a pachychoroid. Therefore, the presence of CSCR cannot be excluded based on a normal choroidal thickness. The SFT can be reduced in pachychoroidal diseases through atrophy of the choriocapillaris and Sattler’s layer, resulting in a decrease in total choroidal thickness [4, 5]. Further, also the unaffected fellow eyes exhibited a mean SFT exceeding the cutoff of 300 μm for the diagnosis of pachychoroid. Thus, SFT, as the sum of the thickness of the choriocapillaris, Sattler’s, and Haller’s layers, is more the result of various influencing factors and choroidal changes and less of an independent and direct influencing factor [5]. Additionally, the use of a fixed SFT threshold is limited since it is also strongly influenced by factors such as axial length and age which were not controlled for in this study. Therefore, while an increasing SFT was found to correlate with an increasing likelihood of CSCR affection, the SFT alone should not be interpreted as a definitive biomarker on its own.

Pachychoroidal vessels were observed frequently in eyes affected by CSCR as well as in the unaffected fellow eyes. The comparison between eyes with CSCR and their fellow eyes without CSCR did not reveal a significant increase in pachyvessels in eyes with CSCR. This finding aligns with current research [10]. Unlike pachyvessels, the presence of intervortex venous anastomoses was significantly more frequently observed in eyes affected by CSCR. This observation could suggest that structural remodeling at the watershed zones may contribute to the pathophysiology of fluid leakage. Furthermore, Savastano et al. also described significant differences in the configuration of the Haller’s layer vessels within the central watershed zone in eyes with acute and chronic CSCR compared to healthy eyes but found no significant differences between the vascular configurations of eyes with acute and chronic forms of CSCR [21]. The authors note that these findings may also be influenced by congenital anatomical factors and raise the question of whether such features predispose individuals to CSCR or represent reactive changes [21]. In contrast, recent research of Lee et al. demonstrated significant differences in choroidal vessel density and the constitution of intervortex anastomoses on ultra-widefield indocyanine green angiography images between eyes with acute and chronic CSCR activity, suggesting that the choroidal vascular architecture also evolves over time with disease progression [22].

Although the occurrence of CSCR is proven to be influenced by systemic factors [16, 17], this study found that CSCR clinically occurs more often as a unilateral rather than bilateral ocular disease. This finding also aligns with current research [1]. While the clinical diagnosis of CSCR bases on the occurrence of subretinal fluid and serous pigment epithelium detachment, pachychoroidal alterations (such as increased subfoveal choroidal thickness and pachyvessels) appear to occur with nearly equal frequency in unaffected fellow eyes. This strongly suggests the involvement of the fellow eye in the disease process, even in the absence of clinical disease activity. Evidently, choroidal thickness and pachyvessels indicate systemic involvement without representing the decisive step in clinical disease manifestation. In contrast, the collapse of the central watershed through the occurrence of intervortex venous anastomoses appears to be significantly associated with the occurrence of the clinical manifest CSCR.

Nonetheless, this study cannot establish causality. Whether intervortex anastomoses lead to disease affection or are a secondary adaptation remains to be clarified in longitudinal studies. Also, dynamic confirmation of venous flow was not feasible with the conducted imaging protocols, and the use of both eyes from bilateral cases as independent observations in statistical analysis could introduce inter-eye correlation, which may bias effect estimates.

This study demonstrates that OCTA is comparable to ICGA, the standard for choroidal vessel imaging, in identifying intervortex venous anastomoses and pachyvessels in patients with CSCR. OCTA even shows advantages in certain cases while providing superior image quality and vessel delineation, likely due to less impairment caused by dye diffusion and masking. This suggests that OCTA has the potential to serve as a reliable non-invasive imaging modality for evaluating choroidal vasculature in patients with CSCR. Besides not requiring dye injection, OCTA offers time-saving benefits by eliminating the need for repeated imaging over a defined period.

Fluorescein angiography (FA) contributes valuable information especially to the clinical assessment of CSCR by revealing characteristic leakage patterns, such as the “smokestack” or “inkblot” signs, which are indicative of active fluid leakage through defects in the retinal pigment epithelium (RPE) [2]. In this study, FA findings were not used as criteria to define CSCR, as the presence of subretinal fluid and serous PED on OCT were prioritized for its direct anatomical depiction. Nevertheless, FA remains a valuable modality, particularly for detecting leakage, assessment of disease activity and guiding treatment [6, 19].

A hyperopic shift of patients with CSCR due to subretinal fluid and detachment of the retinal pigment epithelium is described in literature [23, 24]. However, no significant hyperopic shift attributable to CSCR was detectable, neither in the analysis of the entire study population nor in the separate analysis of the patients with unilateral CSCR. Therefore, an initial indication of and CSCR through determination of refractive errors in clinical routine is not feasible based on this data.

The male-to-female ratio and the significantly lower age of the male patients compared to the female patients in this study population are consistent with current epidemiological research of CSCR in Caucasian populations [25, 26]. While Gender did not appear to be a risk factor for bilateral CSCR, the BCVA of female patients affected by CSCR was significantly worse than that of men affected by CSCR. The visual impairment in female CSCR patients appears to be more severe than in male patients with CSCR. However, further research is needed to investigate potential causality and rule out confounding factors.

Finally, as a single-center study, generalizability is limited, and potential selection bias must be considered. Future multicenter and longitudinal studies are warranted to validate our findings and further investigate the clinical role of intervortex venous anastomoses.

## Conclusion

This study reveals a significant correlation between intervortex venous anastomoses crossing the central watershed and CSCR affection suggesting that intervortex anastomoses could play a crucial role in the development of the clinical manifest disease with subretinal fluid or serous RPE detachment. Moreover, in this study, optical coherence tomography angiography (OCTA) emerged as an alternative to indocyanine green angiography (ICGA) imaging in CSCR diagnosis, offering a non-invasive and time-saving approach without the need for dye injection and repeated imaging.
